# Supplementary material for: Knowledge and Perceptions of Final-Year Nursing Students Regarding Antimicrobials, Antimicrobial Resistance, and Antimicrobial Stewardship in South Africa: Findings and Implications to Reduce Resistance
Source: Antibiotics (Basel). 2023 Dec 16;12(12):1742. doi: 10.3390/antibiotics12121742 (PMC10740495; doi:10.3390/antibiotics12121742)
Supplement: Supplementary file 1 [file antibiotics-12-01742-s001.zip › antibiotics-2750782-supplementary.pdf]

Supplementary Table S1 – Explanation and Questionnaire

1) Letter and Explanation

TITLE OF RESEARCH PROJECT: The knowledge and perceptions of final year nursing students, attending universities in Gauteng, on antimicrobials, antimicrobial resistance and antimicrobial stewardship.

We kindly invite you to participate in a research study by completing this online questionnaire. Participation is ***voluntary***, and you may decide at any time to stop completing the questionnaire. We will not record your email address or IP number.

Since nurses play a crucial role in antimicrobial prescribing, preventing antimicrobial resistance (AMR) and implementing antimicrobial stewardship (AMS) programmes, their training regarding antimicrobials, AMR and AMS are of utmost importance. Participating in this study will help us to assess the above-mentioned factors, since it is necessary for pharmacists to realise the knowledge, attitude and practice of nurses regarding antimicrobials in order to fulfill their role as educators. The research is conducted by Elisma Pieterse, a post-graduate student at the School of Pharmacy, Sefako Makgatho Health Sciences University.

The aim of the study is to assess the knowledge, attitude and practice of final year nursing students on antimicrobials, AMR and AMS. All final year students studying Bachelor of Nursing programmes at one of the six universities in Gauteng offering this programme will be invited to take part in the study. It is anticipated to enroll 298 final year students. Students studying to obtain a diploma or higher certificate in Nursing will be excluded from the study.

When participating in the study, participants will have to answer the questions in the questionnaire to the best of their ability, truthfully and thoroughly. The questionnaire will be once-off thus can only be completed once per participant. The questionnaire will consist of 45 questions, using a Likert-scale to test perception and questions that have different response options to select from (multiple choice) in order to assess participants' knowledge regarding antimicrobials, AMR and AMS and the level of training on these matters. Participating in this study gives no direct benefits to the participant.

Different types of questions tend to average themselves out in most questionnaires and a questionnaire containing 20 to 30 questions is expected to take ten minutes to complete. This however is still to be confirmed during the pilot study but can be expected to take between five to 15 minutes.

There are no direct benefits for participants in this study. All data collected will be captured on a reliable and approved collection template. The data collected will be synthesized by Googles Forms® that can be exported and downloaded to Microsoft Office Excel® for an in-depth analysis. The data will be verified by a statistician to ensure accuracy and reliability of the data analysis. With the email distribution of the questionnaires, Bcc lists will be used so that the 'reply all' function cannot be used by potential participants. Confidentiality through providing anonymity and de-linking any personal identifiable information. The Principal Investigator of this study **Elisma Pieterse** can be contacted at **(083) 579 9746, elismapieterse20@gmail.com** if you have any questions about this study or encounter any problems.

The study will be conducted according to ethical guidelines and principles of the Declaration of Helsinki (2013) and guided by the Department of Health Ethics in Health Research: Principles, Processes and Structures, 2nd Edition (2015). The study has received approval from the Sefako Makgatho University Research Ethics Committee and may be contacted at (012) 521 5617 should there be any concerns regarding the conduct of the study or any complaint.

Your participation is very valuable and appreciated.
'By completing this online questionnaire, I confirm that I am over the age of 18 years, and consent to participate in this study. I have read and understood all the above information provided, and understand that once the survey is submitted, I will no longer be able to withdraw since responses cannot be linked back to me. I participate knowing that my answers will remain confidential and that my identity will not be revealed. I am also aware that the information from this survey may be used for research publication and presentation.'

Ms. Elisma Pieterse | Ms. D Engler (supervisor) | Prof S Bezuidenhout (co-supervisor)

2) Online questionnaire
SECTION 1 OF 6

The knowledge and perceptions of final year nursing students, at universities in Gauteng, on antimicrobials, antimicrobial resistance and antimicrobial stewardship

We kindly invite you to participate in a research study by completing this once-off online questionnaire. Participation is voluntary, and you may decide at any time to stop completing the questionnaire. We will not record your email address or IP number.

Nurses play a crucial role in antimicrobial prescribing, preventing antimicrobial resistance (AMR) and implementing antimicrobial stewardship (AMS) programmes. Therefore, their training regarding antimicrobials, AMR and AMS are of utmost importance. The research is conducted by Elisma Pieterse, a post-graduate student at the School of Pharmacy, Sefako Makgatho Health Sciences University. The aim of the study is to assess the knowledge, attitude and practice of final year nursing students on antimicrobials, AMR and AMS. All final year students studying Bachelor of Nursing programmes at one of the six universities in Gauteng offering this programme will be invited to take part in the study. When participating in the study, participants will have to answer the questions in the questionnaire to the best of their ability and knowledge. The questionnaire consists of 45 questions, including Likert-scale and multiple-choice type questions. There are no direct benefits for participants in this study. The Principal Investigator of this study, Elisma Pieterse can be contacted at (083) 579 9746, elismapieterse20@gmail.com if you have any questions about this study or encounter any problems.

The study will be conducted according to ethical guidelines and principles of the Declaration of Helsinki (2013) and guided by the Department of Health Ethics in Health Research: Principles, Processes and Structures, 2nd Edition (2015). The study has received approval from the Sefako Makgatho University Research Ethics Committee and may be contacted at (012) 521 5617 should there be any concerns regarding the conduct of the study or any complaint.
Your participation is very valuable and appreciated.

By completing this online questionnaire, I confirm that I am over the age of 18 years, and consent to participate in this study. I have read and understood all the above information provided, and understand that once the questionnaire is submitted, I will no longer be able to withdraw since responses cannot be linked back to me. I participate knowing that my answers will remain confidential and that my identity will not be revealed. I am also aware that the information from this survey may be used for research publication and presentation.

○ Yes

○ No

SECTION 2 OF 6

Your institution

Please select at which institute you are currently completing your Bachelor of Nursing studies.
At which institute are you enrolled at?

Tick one of six Universities listed.

SECTION 3 OF 6

General and specific knowledge and awareness of antibiotics

Please select the most appropriate answer.

1. Antimicrobial drugs consist of the following:

   ○ Antibacterial drugs

   ○ Antifungal drugs

   ○ Antiviral drugs

   ○ Antiparasitic drugs

   ○ All of the above

   ○ None of the above

   ○ Not sure

2. Aspirin is an antibiotic:

   ○ Disagree

   ○ Not sure

   ○ Agree

3. Ceftazidime is a 4th generation cephalosporin antibiotic:

   ○ Disagree

   ○ Not sure

   ○ Agree

4. Ceftazidime is a good antibiotic of choice to cover gram-positive organisms:

   ○ Disagree

   ○ Not sure

   ○ Agree

5. Antibiotics are used to treat flu symptoms:

- Disagree
- Not sure
- Agree

6. Antibiotics are useful in treating viral infections:

- Disagree
- Not sure
- Agree

7. Antibiotics are indicated to reduce any kind of pain and inflammation:

- Disagree
- Not sure
- Agree

8. Antibiotics can cause secondary infections after killing good bacteria present in our body:

- Disagree
- Not sure
- Agree

9. Erythromycin is a macrolide antibiotic:

- Disagree
- Not sure
- Agree

10. Antibiotics can cause allergic reactions:

- Disagree
- Not sure
- Agree

11. Patients may stop the use of antibiotics as soon as they feel better:

- ○ Disagree
- ○ Not sure
- ○ Agree

12. Two different types of antibiotics may not be prescribed for simultaneous use:

- ○ Disagree
- ○ Not sure
- ○ Agree

13. Antibiotics should always be prescribed as prophylaxis to prevent future infections:

- ○ Disagree
- ○ Not sure
- ○ Agree

14. Antibiotics cannot treat Human Papilloma Virus (HPV):

- ○ Disagree
- ○ Not sure
- ○ Agree

15. Antibiotics are overused nationally in healthcare:

- ○ Disagree
- ○ Not sure
- ○ Agree

16. Clavulanic acid is given with amoxicillin (co-amoxiclav) in order to decrease inflammation at the site of infection:

- ○ Disagree
- ○ Not sure
- ○ Agree

SECTION 4 OF 6

Perception and knowledge regarding antimicrobial resistance
Please select the most appropriate answer(s):
17. The term "antimicrobial resistance" refers to:

- ○ Patients not wanting to drink their medication thus, they are resistant to using it.
- ○ Antibiotics not working to contain or kill the bacteria as effectively as it previously did.
- ○ Bacteria not being able to infect a human host, the human is resistant to bacterial infections.

18. Which of the fallowing factors contribute towards antimicrobial resistance? (can select more than one): [Checkbox]

- ○ Overuse of antibiotics.
- ○ Use of a broad-spectrum antibiotic when a narrow spectrum is available.
- ○ Poor hand wash practice.
- ○ Poor patient adherence.
- ○ Substandard quality of antibiotics.
- ○ Sub-therapeutic dosing of antibiotics.
- ○ Poor patient counselling.

19. The following interventions can help combat antimicrobial resistance: (you can select more than one): [Checkbox]

- ○ Reduction in antibiotic use.
- ○ Education on antimicrobial therapy for prescribes.
- ○ Antimicrobial usage policies.
- ○ Establish national antimicrobial resistance surveillance.
- ○ Stop antibiotic use completely.
- ○ Development of institutional guidelines for antimicrobial use.
- ○ Take all antimicrobial agents (drugs) off the market.

20. To what extent do you relate with the following statement: strong knowledge of antimicrobials is important in my career:

(A scale of 1-5 is provided for participant to select <u>one option</u>: strongly disagree, disagree, not sure, agree, strongly agree)

- ○ Strongly disagree
- ○ Disagree
- ○ Not sure
- ○ Agree
- ○ Strongly agree

21. To what extent do you relate with the following statement: better use of antimicrobial agents (drugs) will reduce problems with antimicrobial resistance:

- ○ Strongly disagree
- ○ Disagree
- ○ Not sure
- ○ Agree
- ○ Strongly agree

22. To what extent do you relate with the following statement: inappropriate use of antimicrobials can harm patients:

- ○ Strongly disagree
- ○ Disagree
- ○ Not sure
- ○ Agree
- ○ Strongly agree

23. To what extent do you relate with the following statement: antimicrobial resistance is a worldwide problem:

- ○ Strongly disagree
- ○ Disagree
- ○ Not sure
- ○ Agree
- ○ Strongly agree

24. To what extent do you relate with the following statement: antibiotic resistance happens when a bacterium loses its sensitivity to an antibiotic:

- ○ Strongly disagree
- ○ Disagree
- ○ Not sure
- ○ Agree
- ○ Strongly agree

25. To what extent do you relate with the following statement: inappropriate use of antibiotics causes antibiotic resistance:

- ○ Strongly disagree
- ○ Disagree
- ○ Not sure
- ○ Agree
- ○ Strongly agree

26. To what extent do you relate with the following statement: prescribing a broad-spectrum antibiotic increases antibiotic resistance:

- ○ Strongly disagree
- ○ Disagree
- ○ Not sure
- ○ Agree
- ○ Strongly agree

27. To what extent do you relate with the following statement: Poor infection control practices by healthcare professionals cause the spread of antibiotic resistance:

- ○ Strongly disagree
- ○ Disagree
- ○ Not sure

- Agree
- Strongly agree

28. To what extent do you relate with the following statement: antibiotic stewardship is a phenomenon for which a bacterium gains resistance to an antibiotic:

    - Strongly disagree
    - Disagree
    - Not sure
    - Agree
    - Strongly agree

29. To what extent do you relate with the following statement: exposure to antibiotics appears to be the principal risk factor for appearance of antibiotic-resistant bacteria:

    - Strongly disagree
    - Disagree
    - Not sure
    - Agree
    - Strongly agree

30. To what extent do you relate with the following statement: antibiotic resistance can be minimized by using narrow-spectrum therapy after identification and susceptibility testing of infectious bacteria:

    - Strongly disagree
    - Disagree
    - Not sure
    - Agree
    - Strongly agree

31. To what extent do you relate with the following statement: bacteria may acquire efflux pumps that extrude the antibiotic from the cell:

○ Strongly disagree

○ Disagree

○ Not sure

○ Agree

○ Strongly agree

32. To what extent do you relate with the following statement: improving bacterial diagnostics will allow combating antibiotic resistance:

○ Strongly disagree

○ Disagree

○ Not sure

○ Agree

○ Strongly agree

33. To what extent do you relate with the following statement: todays research will be sufficient to meet the future needs for new antibiotics:

○ Strongly disagree

○ Disagree

○ Not sure

○ Agree

○ Strongly agree

SECTION 5 OF 6

Background knowledge on Antimicrobial Stewardship

Select the most appropriate answer.

34. Are you aware of any antimicrobial stewardship programs in South Africa?

○ Yes

○ No

○ Not sure

35. Do you know what antimicrobial stewardship is?

- Yes
- No
- Not sure

36. Where did you hear about it?

- At the university
- In practice
- Both
- None of the above
- Have not heard of the term

37. Implementation of antimicrobial stewardship programs are not yet essential in South Africa?

- Strongly disagree
- Disagree
- Not sure
- Agree
- Strongly agree

38. Antimicrobial Stewardship involves the fallowing: [Checkbox]

- appropriate selection of an antimicrobial agent (drug).
- appropriate dosing of antimicrobial agents (drugs).
- appropriate selection of a route of administration.
- the study of antibiotics.
- selection of an appropriate duration of antimicrobial therapy.

SECTION 6 OF 6

Perception about training on antibiotics received in the nursing degree

Select the most appropriate answer.

39. I have had sufficient nursing education to select the best antibiotic for a specific infection.

- Disagree
- Not sure

○ Agree

40. I have had sufficient education to select an appropriate regimen (dose, route, frequency) of antibiotic therapy.

○ Disagree

○ Not sure

○ Agree

41. I have had sufficient education to understand the mechanism of antibiotic resistance.

○ Disagree

○ Not sure

○ Agree

42. Strong knowledge of antibiotics is important in my nursing career.

○ Disagree

○ Not sure

○ Agree

43. I would like more education on the appropriate use of antimicrobials.

○ Yes

○ No

44. I would like more education on antimicrobial resistance.

○ Yes

○ No

45. Rate how important you consider the role of a nurse in combating antimicrobial resistance:

○ Strongly disagree

○ Disagree

○ Not sure

○ Agree

- ○ Strongly agree

Thank you for your participation, your answers have been recorded.
